# Correlates of metabolic syndrome in people with chronic spinal cord injury

**DOI:** 10.1007/s40618-023-02298-8

**Published:** 2024-01-29

**Authors:** F. Di Giulio, C. Castellini, S. Palazzi, D. Tienforti, F. Antolini, G. Felzani, M. Giorgio Baroni, A. Barbonetti

**Affiliations:** 1 Andrology Unit, Department of Clinical Medicine, Life, Health and Environmental Sciences, University of L’Aquila, 67100 Coppito, L’Aquila, Italy; 2 Spinal Unit, San Raffaele Sulmona Institute, Sulmona, Italy; 3 Neuroendocrinology and Metabolic Diseases, IRCCS Neuromed, Pozzilli, Italy

**Keywords:** Cardiovascular risk, Insulin resistance, Obesity, Pain, Paraplegia, Physical activity

## Abstract

**Purpose:**

We aimed at identifying clinical risk factors or early markers of metabolic syndrome (MetS) in people with spinal cord injury (SCI) that would facilitate a timely diagnosis and implementation of preventive/therapeutic strategies.

**Methods:**

One hundred sixty-eight individuals with chronic (> 1 year) SCI underwent clinical and biochemical evaluations. MetS was diagnosed according to modified criteria of the International Diabetes Federation validated in people with SCI. Wilcoxon rank-sum test and *χ*^2^ test were used to compare variables between groups with and without MetS. Multiple logistic regression analysis was performed to reveal independent associations with MetS among variables selected by univariate linear regression analyses.

**Results:**

MetS was diagnosed in 56 of 132 men (42.4%) and 17 of 36 women (47.2%). At univariate regression analyses, putative predictors of MetS were an older age, a higher number of comorbidities, a lower insulin-sensitivity, the presence and intensity of pain, a shorter injury duration, a poorer leisure time physical activity (LTPA) and an incomplete motor injury. At the multiple logistic regression analysis, a significant independent association with MetS only persisted for a poorer LTPA in hours/week (OR: 0.880, 95% CI 0.770, 0.990) and more severe pain symptoms as assessed by the numeral rating scale (OR: 1.353, 95% CI 1.085, 1.793).

**Conclusion:**

In people with chronic SCI, intense pain symptoms and poor LTPA may indicate a high likelihood of MetS, regardless of age, SCI duration, motor disability degree, insulin-sensitivity and comorbidities.

## Introduction

Life expectancy of spinal cord-injured people has improved markedly in recent decades due to improvements in clinical care of the acute post-injury phase. [1]. Thus, many more patients than in the past survive the acute phase and enter the chronic phase of spinal cord injury (SCI), that is, the stage that begins 1 year after the injury. In that neurologically stable condition, direct and indirect permanent consequences of neurological damage and disability result in systemic comorbidities that unfavorably impact quality of life and increase cardiovascular mortality. The deep anthropometric and body composition changes following SCI strongly contribute to the increased cardiovascular morbidity and mortality [2].

Lack of neurotrophic input of motor nerve projections results in muscle wasting below the injury level up to sarcopenia [3, 4], leading to reduced energy expenditure [5]. On this basis, in people with SCI, body energy balance is positive, as energy intake easily exceeds energy expenditure [6], resulting in increased fat mass, largely distributed at the visceral level [7]. Visceral fat is very sensitive to lipolytic stimuli; therefore, its accumulation is accompanied by an increased release of non-esterified fatty acids in the blood circulation and their deposition into muscle cells and liver [8]. This could explain the very high prevalence of non-alcoholic fatty liver disease (NAFLD) [9–11], as well as the intramuscular fat accumulation [12], peculiar to people with SCI. These pathogenetic processes generate insulin resistance that unfavorably affects glucose and lipid metabolism [13]. The constellation of visceral obesity and its correlates, including insulin resistance, glycemic dysregulation, dyslipidemia, and hypertension has been referred to as “metabolic syndrome” (MetS), a cluster of clinical and metabolic features that increase the cardiovascular risk [8].

In people with SCI, however, the diagnosis of MetS is challenged by the same alterations in body composition that promote its onset. Because of muscle atrophy, BMI underestimates obesity [14, 15] and spinal cord-injured people with a BMI value > 22 kg/m^2^ should be considered as being at high risk for obesity [16]. To further complicate this issue, waist circumference measurement might overestimate visceral fat due to anterior abdominal wall muscle laxity. In addition, especially for injuries above T6, autonomic dysregulation causes blood pressure instability that makes hypertension an unreliable marker of MetS. For these reasons, widely validated diagnostic criteria of MetS in the general population [17, 18] are not reliable in people with SCI for whom modified International Diabetes Federation (IDF) criteria have been proposed [19, 20] with the inclusion of a BMI ≥ 22 kg/m^2^ as a surrogate marker of obesity suitable for SCI [15, 21, 22]. Nevertheless, given the diagnostic challenges, in many people with SCI, MetS may be identified late or go completely undetected. Failure or delay in taking appropriate lifestyle measures and treatments places these individuals at high risk for cardiovascular morbidity and mortality. Identifying clinical variables that could represent risk factors for MetS or its early markers would facilitate a timely diagnosis, allowing the implementation of necessary preventive and therapeutic strategies.

On this basis, the present study aims to identify lifestyle and clinical factors independently associated with MetS in people with chronic SCI.

## Patients and methods

### Design and study population

One hundred sixty-eight consecutive patients (132 men and 36 women; mean age, 54.7 ± 17.2 years) admitted to a routine rehabilitation program because of traumatic SCI were included in this retrospective study. All patients had a documented history of clinically and neurologically stable SCI for more than 1 year. No patient had acute illness hindering the rehabilitative program.

The study was approved by the Ethics Committee of L’Aquila and Teramo provinces, Italy (Approval Code: 11/CE/15 of May 7, 2015) and all enrolled subjects signed an informed consent.

### Clinical evaluations

Chronic medical comorbidities were assessed using a Web-based calculator (https://www.mdcalc.com/calc/3917/charlson-comorbidity-index-cci) of the age-adjusted Charlson Comorbidity Index (CCI). This tool assigns a weighted score to different medical conditions according to both the relative risk of 1-year mortality and the patient's age. The scores are then summed to provide an overall weighted comorbidity index [23].

Patients underwent a thorough neurological examination according to the guidelines of the International Standards for Neurological Examination and Functional Classification of Spinal Cord Injury; the level and completeness of the lesion were defined using the American Spinal Injury Association (ASIA) protocol [24]. According to the ASIA scale, patients with complete injury and no sensory or motor function in the most distal sacral segment were classified as A; those with incomplete injury were classified as B, C, or D. Category B referred to incomplete sensory injury; category C referred to incomplete both sensory and motor injury in which more than half of the 10 key muscle pairs had strength < 3 on a scale ranging from 0 to 5; category D indicated incomplete both sensory and motor injury in which at least half of the key muscles had strength ≥ 3.

The Numeral Rating Scale (NRS) was used to assess the presence of pain related to the SCI and to measure its intensity, according to the recommendations of the National Institute on Disability Research [25]. Participants were asked to verbally rank pain on a scale ranging from 0 to 10, with 0 representing the absence of pain and 10 the maximum pain imaginable.

Body weight was measured with patients wearing light clothing, using a professional mechanical scale (Wunder SA BI Srl, Monza, Italy). Height was determined after patients was placed on the bed, with legs straightened, head on Frankfurt plane, and feet in dorsal flexion. Height was measured with an elastic tape by segmentally measuring the distances between heel and knee, knee and hip, and hip and head. Body mass index (BMI) was calculated in kg/m^2^.

Diagnosis of MetS complied with the modified IDF criteria validated in spinal cord-injured people by Gater and al. [19, 20]: BMI ≥ 22 kg/m^2^ plus any 2 or more of the following features: blood pressure ≥ 130/85 mmHg (or on treatment for hypertension), triglycerides ≥ 150 mg/dL (or on treatment for dyslipidemia), high-density lipoproteins (HDL) < 40 mg/dL for men and < 50 mg/dL for women (or on treatment for dyslipidemia), fasting plasma glucose ≥ 100 mg/dL (or previously diagnosed with type 2 diabetes mellitus).

Functional independence in activities of daily living (ADL) was quantified using the Spinal Cord Independence Measure (SCIM). This is a 19-item tool to measure the functional independence degree in performing ADL: SCIM quantifies each function separately, providing a final score from 0 (total dependence) to 100 (total independence) [26].

Leisure Time Physical Activity (LTPA) refers to physical activities that people choose to perform in their free time: in people with SCI, it includes walking/wheeling, and sports played in a gym. The LTPA Questionnaire (LTPAQ) for People with SCI was used to quantify these activities [27]. This tool provides a measure of hours of LTPA performed at each intensity (mild, moderate, and heavy intensity LTPA) over the previous 7 days. As previously reported [28–30], only total score was included in the analyses, as it strongly correlated with mild, moderate, and heavy intensity LTPA sub-scores [29].

### Laboratory examinations

A single morning venous blood sample after at least 12 h of fasting was obtained from each participant between 8:00 and 9:00 a.m. Standard methods and commercial kits (Instrumentation Laboratory Company, Lexington, MA, USA) were used for all the biochemical and hematologic measurements. Insulin resistance was assessed using the homeostatic model assessment of insulin resistance (HOMA-IR), according to the formula: insulin (mU/L) × glucose (m/dL)/405 [31].

### Statistical analysis

Statistical analysis was performed using the R statistical software (version 4.0.4, 2021, The R Foundation for Statistical Computing, Vienna, Austria). After ascertaining the non-normal data distribution with the Shapiro–Wilk test, we used the Wilcoxon rank-sum test (Mann–Whitney U test) to compare variables between subjects with and without MetS. Proportional differences were assessed by the χ^2^ test. Multiple logistic regression analysis was performed to reveal independent associations with MetS among variables selected by univariate linear regression analyses. Variance inflation factors (VIFs) were calculated to check the collinearity assumption: a VIF < 5 was considered to indicate no significant collinearity. Significance of differences in the prevalence of MetS throughout the 1st to the 3rd tertile of increasing pain NRS score and LTPA levels were assessed by χ^2^ test for trend (Mantel–Haenszel test). Statistical significance was accepted when *p* < 0.05.

## Results

A diagnosis of MetS was made in 56 of 132 men (42.4%) and 17 of 36 women (47.2%). Table 1 shows the characteristics of the study population categorized by MetS. Patients with MetS were significantly older and had higher triglycerides and lower HDL levels, higher HOMA-IR, BMI, CCI, systolic and diastolic blood pressure, pain NRS score and poorer LTPA. Moreover, in patients with MetS, the prevalence of complete motor injury was lower, as was the duration of injury (DOI).

**Table 1:** Characteristics of the study population categorized by metabolic syndrome

Characteristics	MetS yes (*n* = 73)	MetS no (*n* = 95)	*p* value
Physiological and life-style variables			
Age (years)	60 (51–70)	49 (34–65)	< 0.0001
Gender, *n* (%)			
Males	56 (77)	76 (80)	0.74
Females	17 (23)	19 (20)	
Current smokers, *n* (%)	18 (25)	40 (42)	0.03
Blood biometric measures			
Total cholesterol (mg/dL)	181 (157–195)	161 (136–188)	0.01
Triglycerides (mg/dL)	161 (115–214)	96 (74–119)	< 0.0001
HDL (mg/dL)	35.5 (30.7–40.0)	43.5 (38.0–50.0)	< 0.0001
LDL (mg/dL)	113 (88–131)	101 (80–119)	0.05
HOMA-IR	2.1 (1.4–2.9)	1.4 (0.8–1.9)	0.0001
Glycemia (mg/dL)	95 (86–102)	83 (77–89)	0.0001
Insulin (µU/mL)	8.9 (6.6–11.8)	6.5 (4.0–9.6)	0.003
Clinical and injury-related variables			
BMI (kg/m^2^)	27.7 (25.7–29.3)	24.5 (22.0–27.5)	< 0.0001
CCI	3 (1–5)	1 (0–3)	< 0.0001
Systolic blood pressure (mmHg)	120 (110–130)	110 (100–117)	< 0.0001
Diastolic blood pressure (mmHg)	76 (70–80)	70 (62–75)	< 0.0001
Neurological level of the lesion, *n* (%)			
Cervical level	22 (30)	34 (36)	0.52
Thoracic-lumbar level	50 (70)	59 (64)	
Lesion completeness, *n* (%)			
Complete motor lesion (AIS A-B)	35 (48)	62 (65)	0.04
Incomplete motor lesion (AIS C-D)	38 (52)	33 (35)	
Functional independence (SCIM score)	47 (25–65)	49 (28–64)	0.79
Numeral Rating Scale (NRS) pain score	5 (3–7)	4 (0–5)	0.004
LTPA (hours/weekly)	5.3 (2.9–9.8)	9.3 (4.8–14.0)	0.0003
DOI (years)	5 (2–10)	9 (3–17)	0.03
Pain medications, *n* (%)			
NSAIDs	12 (16.4)	10 (10.5)	0.37
Acetaminophen	8 (11.0)	7 (7.4)	0.59
Tramadol	8 (11.0)	6 (6.3)	0.43
Gabapentin/pregabalin	23 (31.5)	24 (25.3)	0.47

Data were expressed as median (25th–75th centiles) for continuous variables and as percentages when categorical

*AIS* ASIA (American Spinal Injury Association) Impairment Scale, *BMI* Body Mass Index, *CCI* Charlson comorbidity index, *DOI* duration of injury, *HDL* High-Density Lipoprotein, *HOMA-IR* Homeostatic Model Assessment of Insulin Resistance, *LDL* Low-Density Lipoprotein, *LTPA* leisure time physical activity, *MetS* metabolic Syndrome, *NRS* Numeral Rating Scale, *NSAIDs* Non-Steroidal Anti-Inflammatory Drugs, *SCIM* Spinal Cord Independence Measure

As shown in Table 2, at the univariate regression analyses, putative significant predictors of MetS were an older age, a higher CCI, a higher pain NRS score and HOMA-IR, as well as a shorter DOI, a poorer LTPA and the presence of incomplete motor injury. At the multiple logistic regression analysis, a significant independent association with MetS only persisted for a poorer LTPA and more severe pain symptoms, as indicated by a higher pain NRS score.

**Table 2:** Univariate and multiple logistic regression analyses of putative predictors of MetS

Variables	Univariate regressions		Multivariable regression		
	OR (95% CI)	*p* value	VIF	OR (95% CI)	*p* value
Age (years)	1.040 (1.020; 1.060)	0.0002	2.433	1.016 (0.964; 1.070)	0.557
Smoke	0.577 (0.295; 1.107)	0.102	-	-	-
CCI	1.310 (1.160; 1.50)	< 0.0001	2.297	0.959 (0.647; 1.424)	0.829
DOI	0.960 (0.930; 0.990)	0.048	1.211	0.951 (0.890; 1.008)	0.107
NRS pain score	1.180 (1.050; 1.330)	0.006	1.395	1.353 (1.085; 1.739)	0.011
HOMA-IR	1.62 (1.197; 2.344)	0.005	1.184	1.596 (0.969; 2.983)	0.106
Complete motor lesion (AIS A-B)	0.490 (0.260; 0.910)	0.025	1.134	1.052 (0.323; 3.558)	0.933
LTPA (hours/week)	0.890 (0.820; 0.950)	0.002	1.384	0.880 (0.770; 0.990)	0.037

*AIS* ASIA (American Spinal Injury Association) Impairment Scale, *CCI* Charlson comorbidity index, *CI* confidence interval, *DOI* duration of injury, *HOMA-IR* Homeostatic Model Assessment of Insulin Resistance, *LTPA* leisure time physical activity, *NRS* Numeral Rating Scale, *OR* odds ratio, *VIF* Variance Inflation Factor

**Fig.1:**
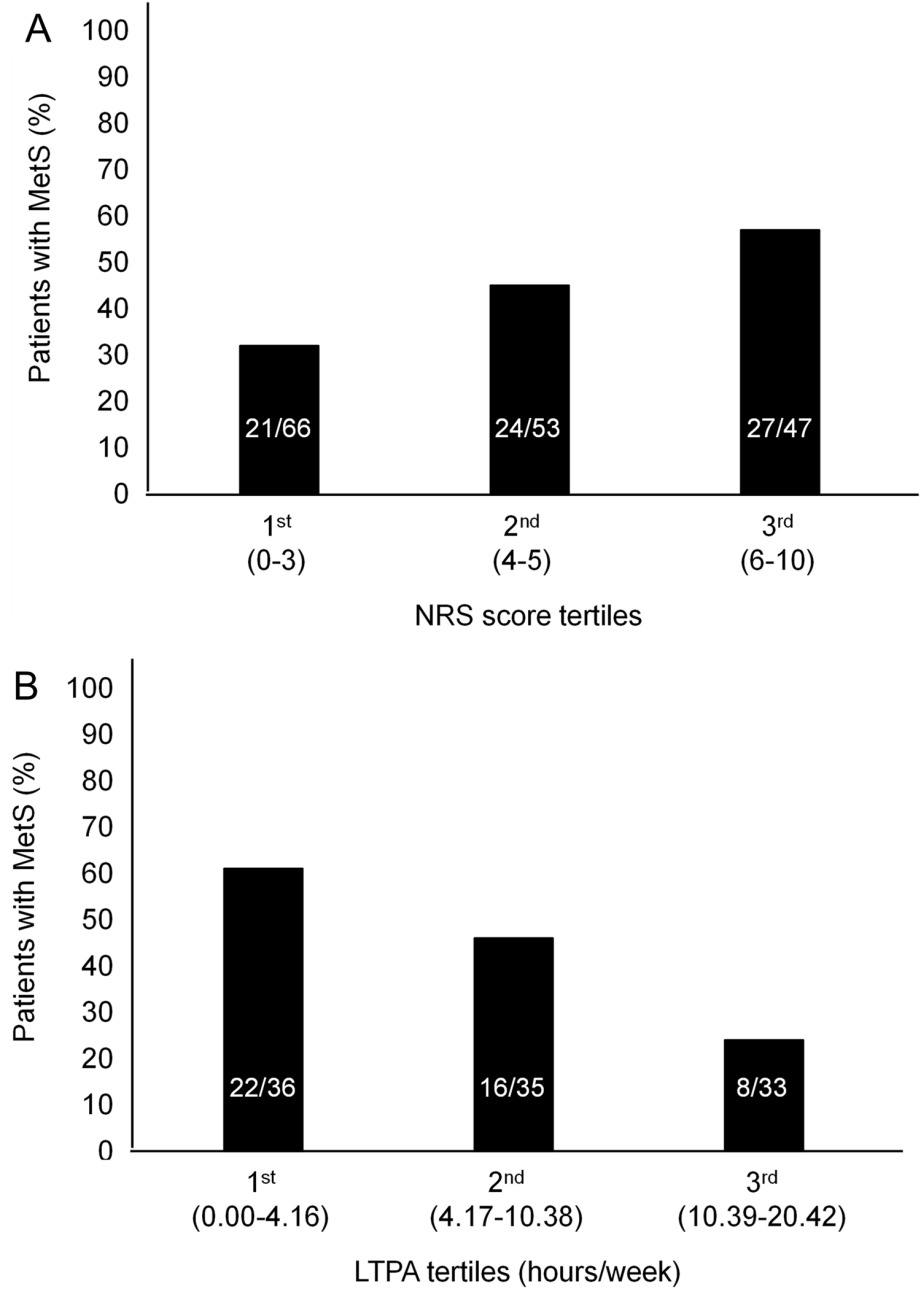
Prevalence of metabolic syndrome (MetS) by **A** pain intensity quantified in tertiles of increasing numeral rating scale (NRS) score and **B** weekly leisure time physical activity (LTPA). Statistical significance for differences among groups of NRS scores, obtained by categorizing data into tertiles: *χ*^2^ test for trend = 7.46, *p* = 0.02; Statistical significance for differences among groups of LTPA, obtained by categorizing data into tertiles: *χ*^2^ test for trend = 9.53, *p* = 0.008)

As the pain NRS score and LTPA level increased from the 1st tertile to the 3rd tertile, the prevalence of MetS significantly increased (χ^2^ test for trend: 7.46, *p* = 0.02, Fig. 1A) and decreased (*χ*^2^ test for trend: 9.53, *p* = 0.008, Fig. 1B), respectively.

## Discussion

Among the many possible determinants and attributes of MetS in people with SCI, this study has narrowed the field to a limited number of variables. The results of univariate regression analyses showed a significant association of MetS with older age, more comorbidities, lower insulin sensitivity, more intense pain symptoms, a poorer physical activity, a lower degree of neuromotor disability, and a more recent clinical history of SCI.

Advancing age is commonly associated with a progressive decrease in physical activity and worsening overall health status due to the onset of comorbidities, including the MetS components [32]. Decreased physical activity, quantified in terms of LTPA in the present study, may contribute directly to the increased incidence of comorbidities, insulin resistance and MetS [2]. An additional factor with a possible pathogenetic role is pain. The prevalence of MetS significantly increased throughout the lowest to the highest tertile of pain NRS score. Neuropathic and/or nociceptive chronic pain symptoms represent a major complaint in people with SCI [33]. Pain may further limit mobility and thus the ability to engage in physical activity: the resulting drop in energy expenditure increases fat accumulation and the risk of developing comorbidities and MetS [2]. Poor physical activity and hypomobility, in turn, could contribute to muscle spasticity that exacerbates pain symptoms [33], thus triggering a vicious circle. The possible mechanisms by which MetS is associated with a lower degree of motor disability and a shorter DOI in our univariate regression analyses remain speculative but could involve lifestyles. In people with recent history of SCI, an incomplete psychological and social-relational adjustment to the disabling condition [34] could result in a propensity to exhibit behavioral correlates of emotional distress [35, 36] at risk for the development of comorbidity, including MetS components. This might occur, paradoxically, with greater likelihood in people with incomplete motor SCI, since, as previously reported [37], spinal cord-injured people with less severe disability degree, have an increased independence in lifestyle and eating habits. In keeping with these findings, in a recent series of 166 Korean patients with chronic SCI, a lower neurological level of injury was a significant and independent predictor of MetS [38]. Indeed, other authors have documented that people with paraplegia exhibited more unfavorable anthropometric features than those with tetraplegia [39].

Obviously, the cross-sectional design of this study does not allow the causal directionality of the associations under investigation to be established, but some information can be inferred from the multiple logistic regression analysis. When all variables selected by the univariate analyses were included in the same multivariable model, only a poorer LTPA and a higher pain score at the NRS exhibited a significant and independent association with MetS. This finding demonstrated that the relationship between pain and MetS is not necessarily mediated by reduced physical activity. Indeed, the association of MetS with poor LTPA and more intense pain symptoms may be complex and bidirectional: on one hand, obesity and comorbidities, components of MetS, may constrain the ability to engage in physical activity; on the other hand, there is preclinical, clinical, and epidemiologic evidence for an association between MetS and sensory peripheral neuropathy independent of prediabetes, diabetes, and glycemic status [40, 41]. The main mediators of this association would be obesity and dyslipidemia [40]. Obesity is the hallmark of MetS and is accompanied by the release into circulation of a large pool of long-chain fatty acids (LCFAs) that penetrate the blood–neurogenic barrier, causing oxidative stress-mediated neuroinflammation [42]. LCFAs alter axonal mitochondrial transport and impair electron chain activity [43]. The resulting mitochondrial dysfunction results in impaired oxidative phosphorylation with reduced adenosine triphosphate (ATP) production and generation of reactive oxygen species (ROS) [44, 45]. The nuclear factor kappa B (NF-kB), a transcriptional factor activated by oxidative stress and hyperglycemia, is at the center of neuroinflammation by MetS [46] as it modulates several downstream pro-inflammatory genes, particularly cyclooxygenase-2 (COX-2) [47]. These mechanisms as whole underlie neuronal and Schwann cell injury that ultimately contributes to MetS neuropathy resulting in chronic pain [40].

Of note, the negative association between MetS and insulin sensitivity was lost in the fully adjusted multiple logistic regression analysis. In keeping with this result, in a recent study by Solinsky et al. [48], although 95 individuals with SCI exhibited a significantly higher prevalence of obesity and lower levels of HDL when compared to 1609 able-bodied controls from the population of National Health and Nutrition Examination Education Survey (NHANES), the rate of insulin resistance, as assessed by HOMA-IR, was similar between the two groups. These findings may reflect the peculiarities of the pathogenesis of MetS in people with SCI. It is possible to speculate that the SCI-related chronic inflammatory state, in addition to contributing to the onset of neuro-pathic pain, may promote at the muscle and adipose tissue level mechanisms of glucose and lipid deregulation underlying MetS, regardless of insulin sensitivity degree. Indeed, although HOMA-IR was significantly higher in patients with MetS than in those without MetS, in both groups, its median values were largely within the normal range. On the other hand, the lack of independent association between MetS and higher HOMA-IR does not seem to depend on a lack of reliability of the latter to identify insulin resistance in people with SCI, since in a recent study by Farkas et al. [49], HOMA-IR and Quantitative Insulin-sensitivity Check Index (QUICKI) were the indices of insulin sensitivity with the best agreement with the intravenous glucose tolerance test in this population.

This study has some limitations. First, the relatively small sample size and the enrollment within a clinical rehabilitation setting. These two peculiarities may limit the generalizability of the results to the community-dwelling spinal cord-injured population. Despite a relatively sized series, the high prevalence of the endpoint (e.g., MetS) allowed the inclusion of a fair number of independent variables in the multiple regression model to adjust analysis for possible major confounders. Nevertheless, a residual confounding effect from unmeasured variables potentially mediating the revealed associations cannot be ruled out. In this light, factors related to lifestyle and/or psychological functioning could play a major role and deserve to be investigated in targeted studies.

In conclusion, in people with chronic SCI, intense pain symptoms and poor physical activity may suggest a high likelihood of MetS, regardless of age, DOI, degree of motor disability, insulin sensitivity and comorbidities. Given the challenges in diagnosing MetS in this population [2], pain and physical inactivity can help health care providers to identify the most at-risk individuals early so that all necessary measures for prevention and treatment of cardiovascular implications can be implemented.

## Data Availability

The datasets generated during and/or analyzed during the current study are available from the corresponding author on reasonable request.
